# Supplementary figures and images for: Sphingosine‐1‐Phosphate Receptor 1 Regulates Competition Dependent Astrocyte Morphogenesis and Tiling in Murine Cortex
Source: Glia. 2026 Jun 1;74(8):e70177. doi: 10.1002/glia.70177 (PMC13366563; doi:10.1002/glia.70177)

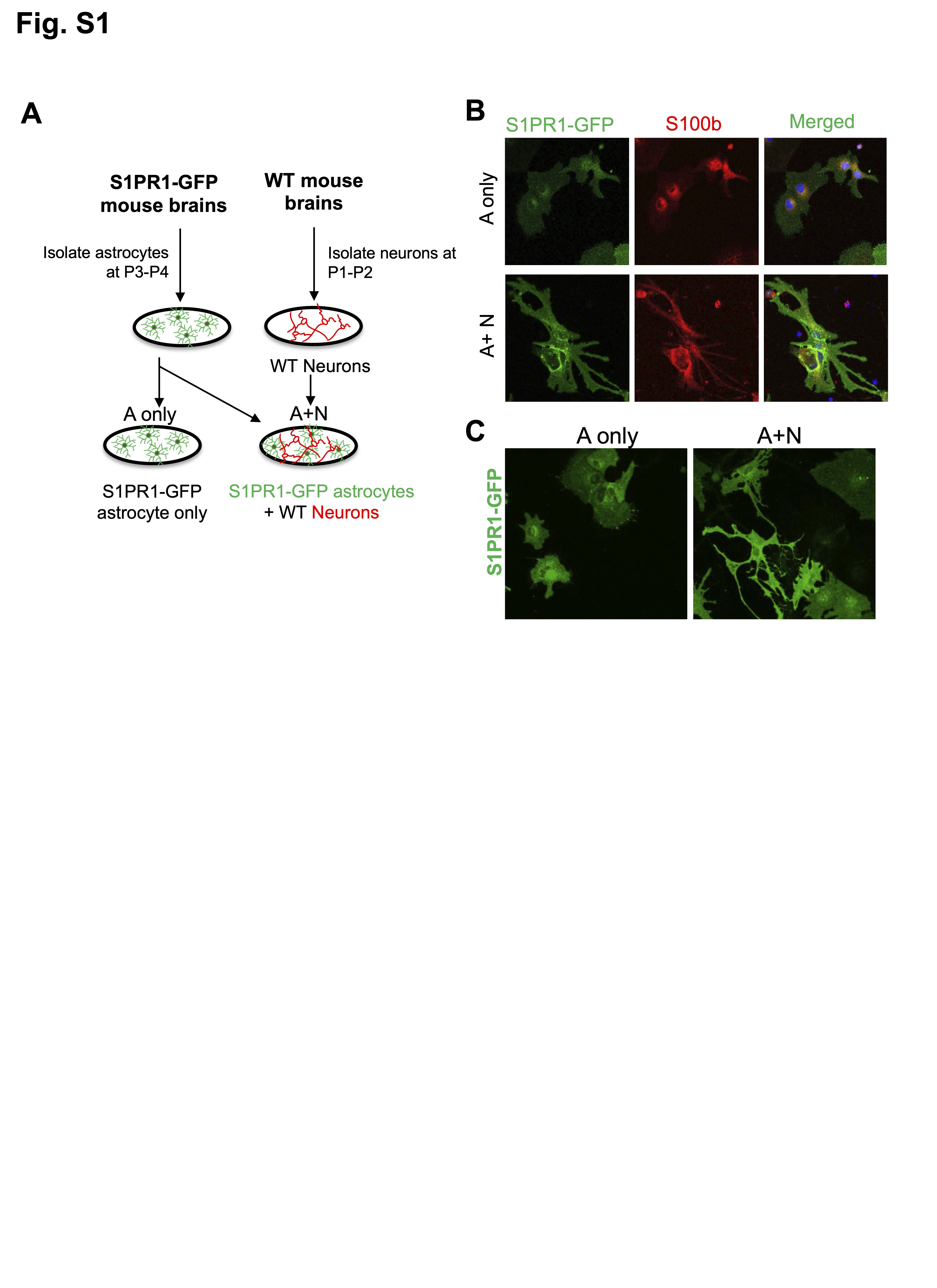

Supplement: Supplementary file 1 — Figure S1: Astrocyte, neuron isolation and coculture from S1PR1‐GFP reporter mice and WT mice respectively to study expression and function of S1PR1. (A) Schematics of isolation and culture of astrocyte and neurons in vitro. (B) Purity of astrocyte cultures and expression of S1PR1‐GFP (green) appears plasma membrane and stained with astrocyte marker S100b (red) in astrocyte only and A + N cocultures. (C) S1PR1‐GFP expressing astrocytes showed two population of cells bright with complex morphology versus dim and roundish less complex cellular morphology. [file GLIA-74-0-s001.jpg]

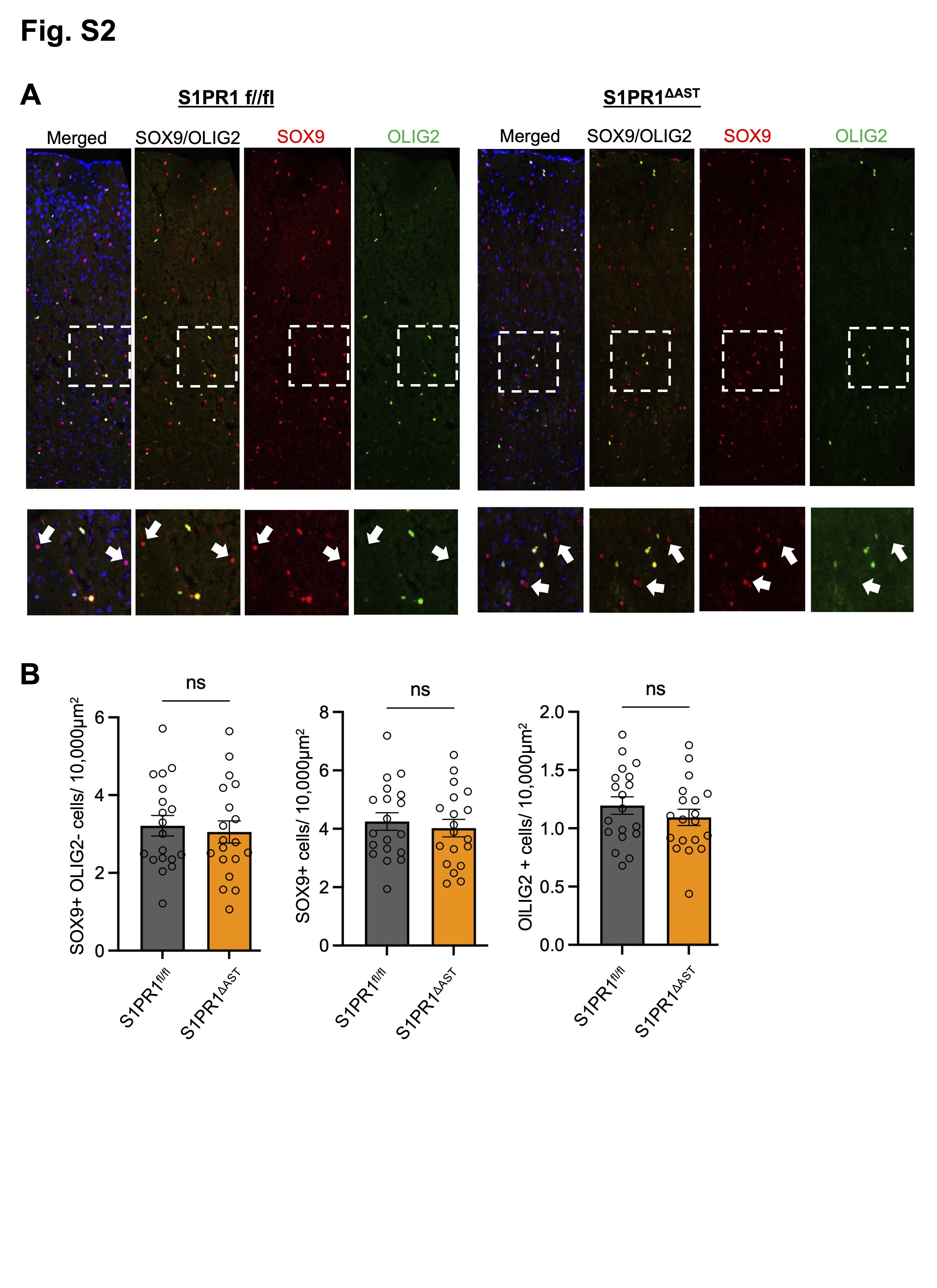

Supplement: Supplementary file 2 — Figure S2: Astrocyte numbers as quantified by SOX9+ and Olig2− remain unchanged in S1PR1ΔAST mice. (A) Representative confocal 20× images stained with SOX9 (red) OLIG2 (green) and Dapi (blue) from somatosensory cortices of S1PR1ΔAST or littermate control mice at P30. Boxed area is zoomed in and presented at the lower panels. Arrowheads indicate SOX9+OLIG2− astrocytes. (B) Quantification of SOX9+OLIG2− astrocytes (left), SOX9+ cells (middle) and OLIG2+ cells (right) represented as the cells/10,000 μm2 showing no changes in astrocyte numbers. Data represents the mean ± SEM of 19 and 19 images n = 3 and 3 mice per group. ns = non‐significant, Unpaired Welch's t‐test. [file GLIA-74-0-s003.jpg]

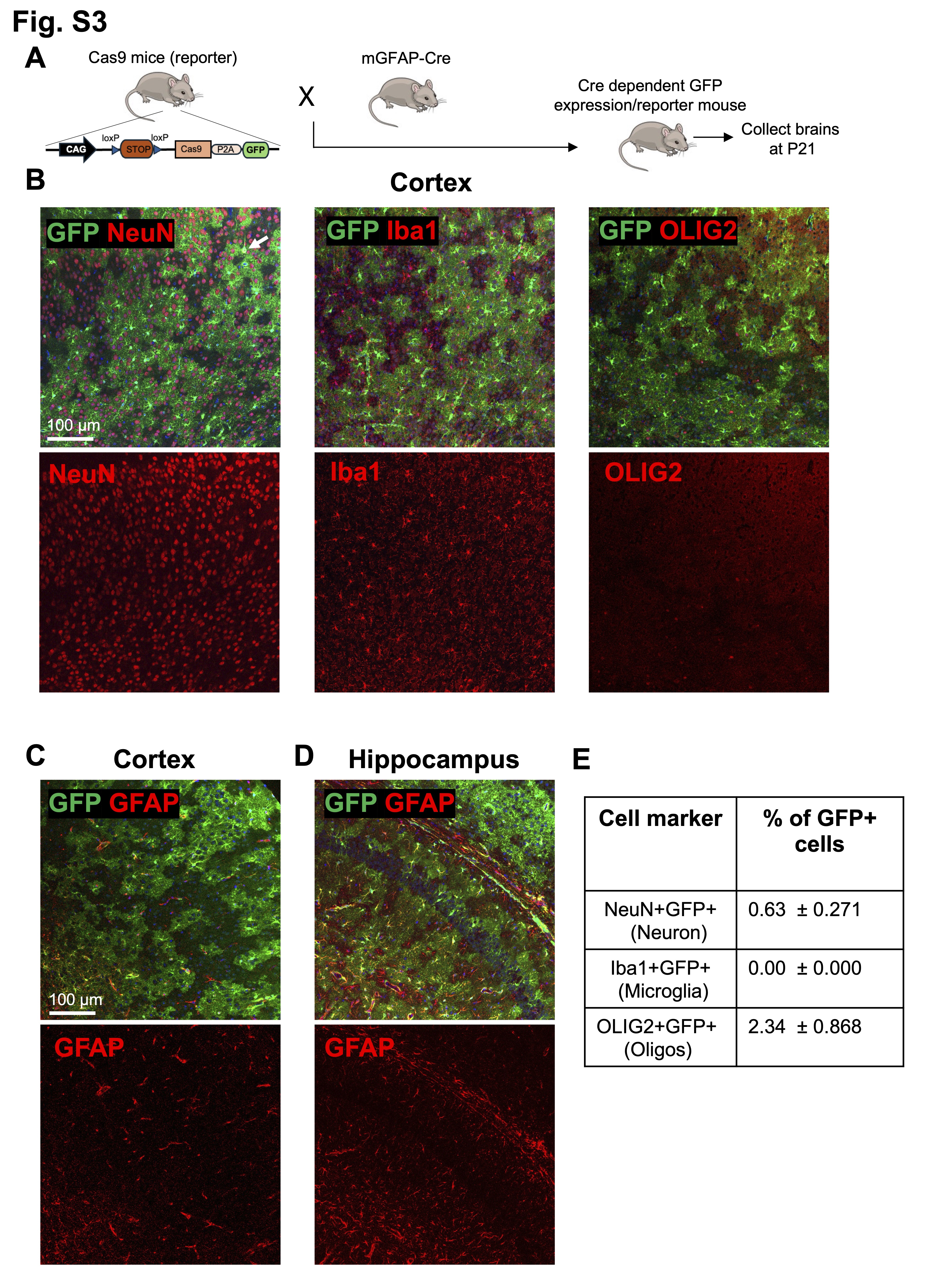

Supplement: Supplementary file 3 — Figure S3: GFAP‐Cre dependent GFP reporter mouse display minimal reporter expression in non‐astrocytic cells. (A) Schematics to show our breeding strategy to generate Cre dependent reporter mouse line by crossing our GFAP‐Cre (no flox) with Lox‐STOP‐Lox‐Cas9‐P2A‐GFP mouse line. (B) Representative confocal 20× images stained with GFP (green) and costained with either neuronal marker NeuN (red) (arrow indicates a NeuN+ GFP+ neuron), or microglial marker Iba1 (red), or Oligodendrocyte lineage marker OLIG2 (red) or (C) astrocyte marker GFAP (red) from P24 cortices or Hippocampus (only GFAP/GFP (D)). (E) Table with quantification of GFP+NeuN+, or GFP+Iba1+ or GFP+OLIG2+ cells as % of GFP labeled cells. Data represents the mean ± SEM of 9–11 images from n = 3 mice. [file GLIA-74-0-s004.jpg]

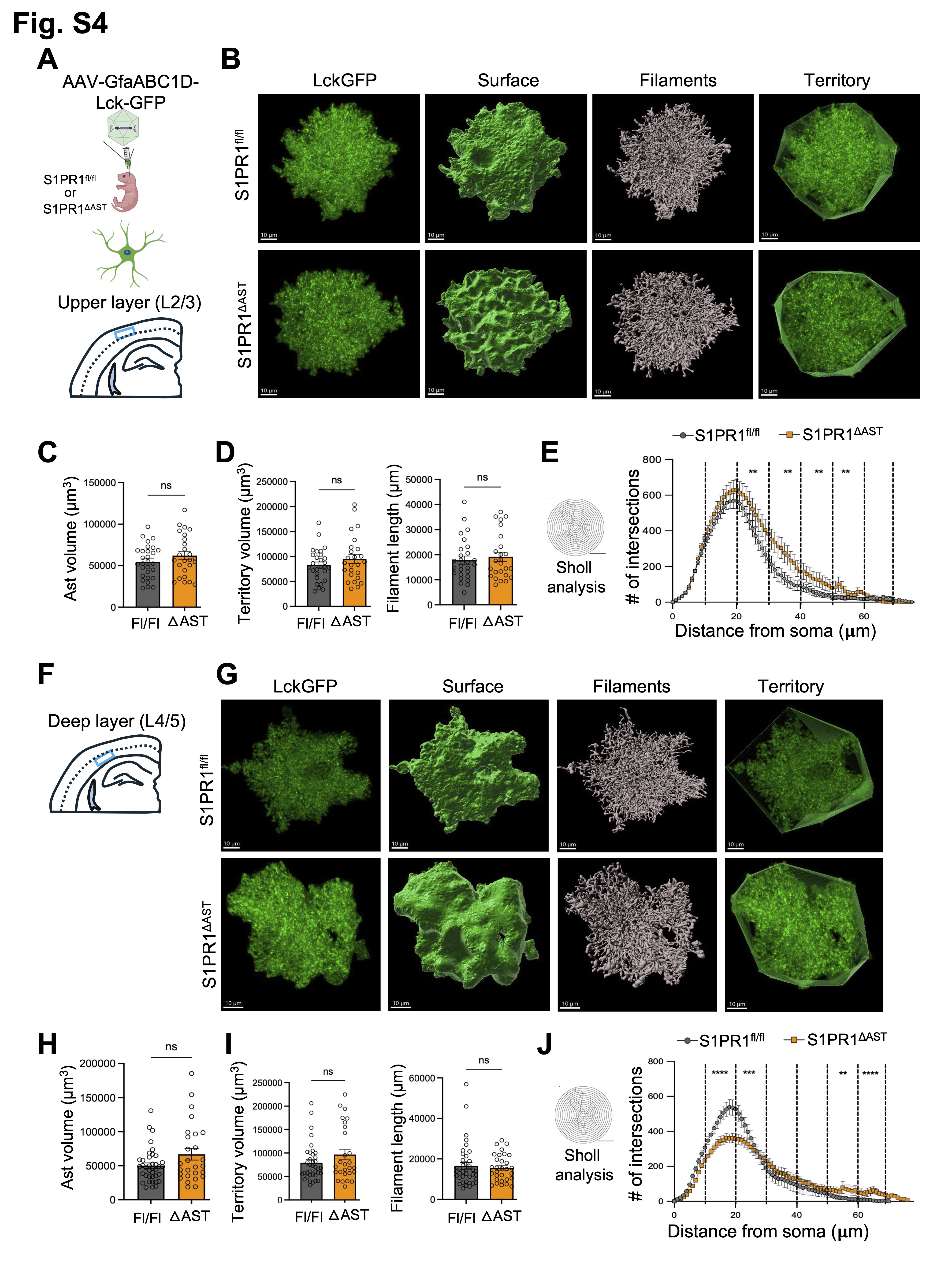

Supplement: Supplementary file 4 — Figure S4: Membrane‐targeted Lck‐GFP labeled astrocytes from S1PR1ΔAST mice exhibit complexity changes. (A) Schematics showing AAV‐based membrane labeling of L2‐3 astrocytes in vivo by Lck‐GFP. (A–F) Sparsely labeled astrocytes from L2‐3 somatosensory cortices were imaged and analyzed. (B) Representative, confocal L2‐3 membrane targeted‐GFP filled astrocytes (left panel) and IMARIS rendered surface traces (right panels) (C) IMARIS rendered filament traces and astrocyte territory. (D) Quantification of astrocyte volume from Surface renderings generated using IMARIS. (E) Quantification of astrocyte territory volume (left) and filament length from filament traces generated using IMARIS. (F) Sholl analyses of IMARIS rendered filament traces of Lck‐GFP labeled astrocytes from S1PR1ΔAST and littermate controls. Data represents the mean ± SEM of 25–28 astrocytes from n = 3 mice per group. *p < 0.05, **p < 0.01, ns = non‐significant, Unpaired Welch's t‐test. (G–L) Sparsely labeled astrocytes from L4‐5 somatosensory cortices were imaged and analyzed. (H) Representative, confocal L4‐5 membrane targeted‐GFP filled astrocytes (left panel) and IMARIS rendered surface traces (right panels) (I) IMARIS rendered filament traces and astrocyte territory. (J) Quantification of astrocyte volume from Surface renderings generated using IMARIS. (K) Quantification of astrocyte territory volume (left) and filament length from filament traces generated using IMARIS. (L) Sholl analyses of IMARIS rendered filament traces of Lck‐GFP labeled astrocytes from S1PR1ΔAST and littermate controls. Data represents the mean ± SEM of 27 and 37 astrocytes from n = 3 mice per group. *p < 0.05, **p < 0.01, ****p < 0.0001, ns = non‐significant, Unpaired Welch's t‐test. [file GLIA-74-0-s002.jpg]

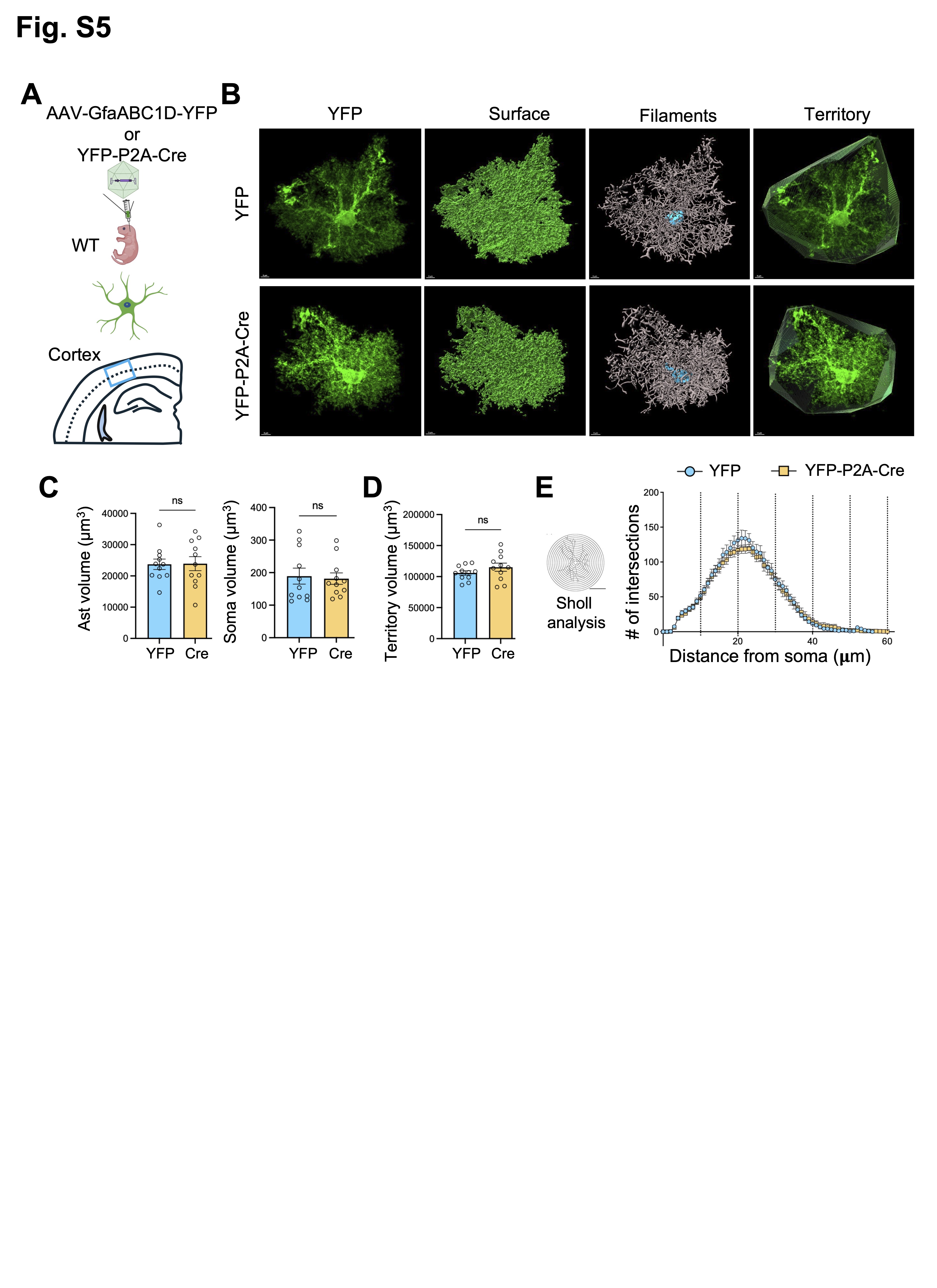

Supplement: Supplementary file 5 — Figure S5: Expression of Cre by AAV does not alter astrocytes morphology. (A) Schematics showing delivery of AAV expressing GfaABC1D‐YFP‐P2A‐Cre or GfaABC1D‐YFP control in wildtype (WT) mouse pups. Sparsely labeled astrocytes were imaged from both upper and deeper somatosensory cortex from P30 pups. (B) Representative confocal images and subsequent IMARIS 3D surface/filament renderings of YFP astrocytes from control or Cre expressing littermates at P30. (C) Quantification of astrocyte volume (left) and soma volume (right) from Surface renderings generated using IMARIS. (D) Quantification of astrocyte territory volume from filament traces generated using IMARIS. (E) Sholl analyses of IMARIS rendered filament traces of astrocytes from Cre.P2A.YFP and YFP only. Data represents the mean ± SEM of 11 and 11 astrocytes from n = 3 mice per group. ns = non‐significant, Unpaired Welch's t‐test. [file GLIA-74-0-s006.jpg]

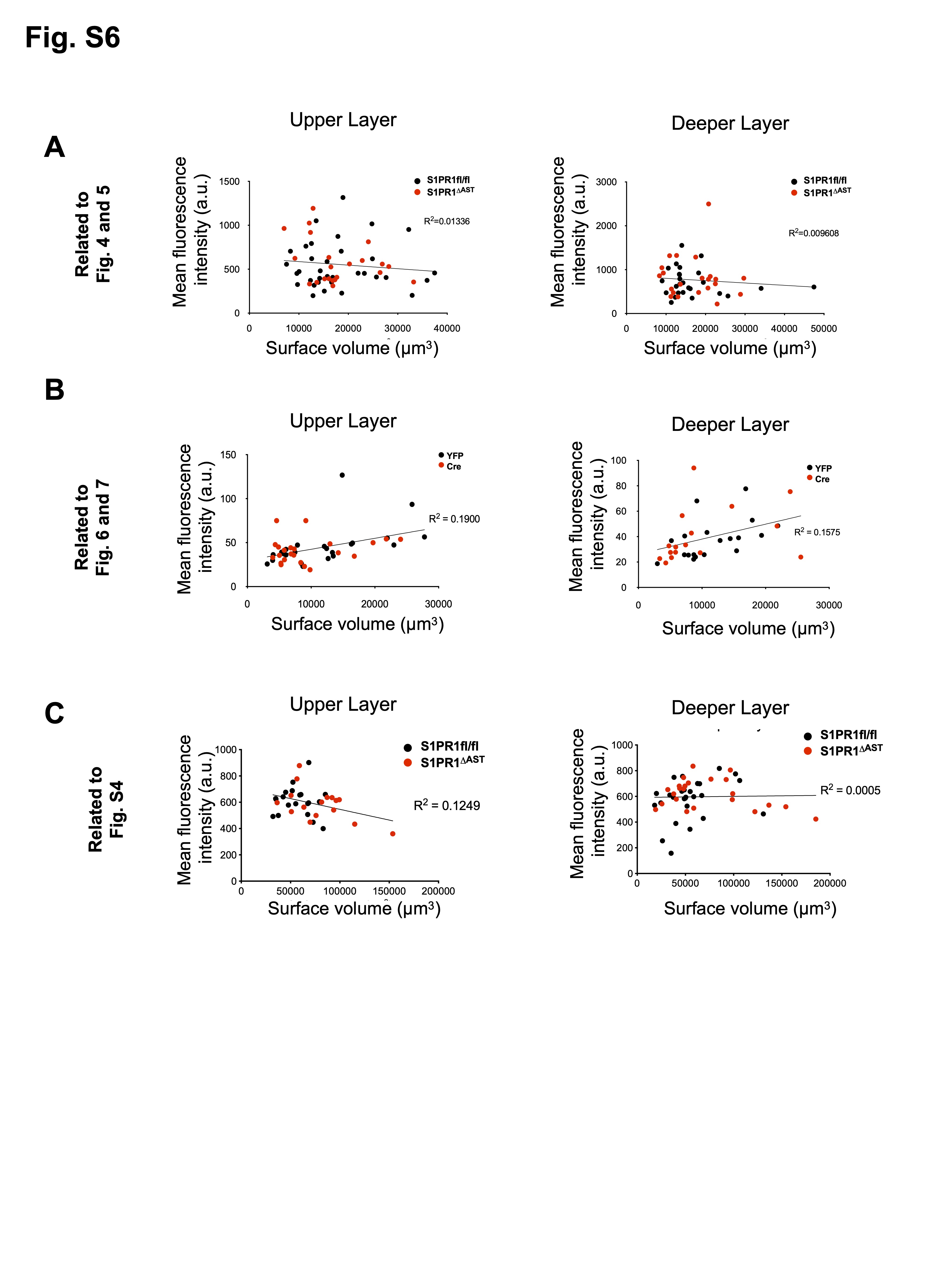

Supplement: Supplementary file 6 — Figure S6: Cell size does not correlate with GFP/YFP intensity. Astrocyte's surface volume was plotted against its own mean fluorescent intensity in a layer‐specific manner from respective experiments A, B and C. Linear regression model was used to fit a line with R squared values. [file GLIA-74-0-s005.jpg]
